# Investigating the Mechanisms Underlying the Low Irradiance-Tolerance of the Economically Important Seaweed Species *Pyropia haitanensis*

**DOI:** 10.3390/life13020481

**Published:** 2023-02-09

**Authors:** Dehua Ji, Yichi Zhang, Bao Zhang, Yan Xu, Kai Xu, Changsheng Chen, Chaotian Xie

**Affiliations:** 1 Fisheries College, Jimei University, Xiamen 361021, China; 2 Fujian Engineering Research Center of Aquatic Breeding and Healthy Aquaculture, Xiamen 361021, China; 3 Key Laboratory of Healthy Mariculture for the East China Sea, Ministry of Agriculture, Xiamen 361021, China

**Keywords:** low irradiance stress, photomorphogenesis, signaling, transcriptomics

## Abstract

*Pyropia haitanensis*, one of the most economically and ecologically important seaweed species, is often exposed to persistent or transient low irradiance (LI), resulting in limited yield and quality. However, the mechanisms mediating *P. haitanensis* responses to LI are largely unknown. In this study, LI-tolerant (LIT) and LI-sensitive (LIS) *P. haitanensis* strains were compared regarding their physiological and transcriptomic changes induced by 1 and 4 days of LI (5 μmol photons/m^2^·s). The results indicated that the inhibition of photomorphogenesis and decreases in photosynthesis and photosynthetic carbon fixation as the duration of LI increased are the key reasons for retarded blade growth under LI conditions. A potential self-amplifying loop involving calcium signaling, phosphatidylinositol signaling, reactive oxygen species signaling, and MAPK signaling may be triggered in blades in response to LI stress. These signaling pathways might activate various downstream responses, including improving light energy use, maintaining cell membrane stability, mitigating oxidative damage, to resist LI stress. Additionally, the LIT strain maintained transcriptional homeostasis better than the LIS strain under LI stress. Specifically, photosynthesis and energy production were relatively stable in the LIT strain, which may help to explain why the LIT strain was more tolerant to LI stress than the LIS strain. The findings of this study provide the basis for future investigations on the precise mechanisms underlying the LI stress tolerance of *P. haitanensis*.

## 1. Introduction

*Pyropia/Porphyra* (also known as *Neopyropia/Neoporphyra*) species are economically and ecologically important mariculture seaweeds. *Pyropia* species are farmed and processed by large seaweed-related industries in East Asian countries, including China, South Korea, and Japan [1]. The consumption of dried seaweed has recently increased worldwide because of the growing awareness of its nutritional benefits, and its cultivation is in line with global sustainability targets [2,3,4]. The economic value of *nori* produced worldwide increased from USD 945.1 billion in 1987 to USD 2319.7 billion in 2017 [5]. Wild *Pyropia/Porphyra* species inhabit the intertidal zone and can survive direct exposure to sunlight at low tide, suggesting that their blades have evolved to become highly tolerant to high irradiance. However, the blades of cultivated *Pyropia/Porphyra* species are often exposed to persistent or transient low irradiance (LI). Because of human activities in coastal waters, including dredging, land reclamation, dock construction, offshore operations, and aquaculture, the seawater in seaweed-cultivation regions has become less transparent. Additionally, continuous rainfall can induce LI stress in cultivated *Pyropia/Porphyra* blades. The detrimental effects of LI stress on *Pyropia/Porphyra* blades result in decreased yield and quality. For example, a recent study demonstrated that compared with the effects of 500 μmol photons/m^2^·s, treatment with 5 days of 10 μmol photons/m^2^·s decreased the *Pyropia haitanensis* blade growth rate by approximately 29% and 37% at a pH of 7.5 and 8.1, respectively [6]. Therefore, investigating the mechanisms mediating the responses of *Pyropia/Porphyra* blades to LI stress may generate useful information for breeding LI-tolerant strains.

LI stress induces numerous metabolic changes in plants by suppressing photosynthesis-related factors and activities and disrupting assimilation and metabolism [7,8,9]. Specifically, the decrease in the photosynthetic rate leads to a decrease in the dry matter content and grain yield in response to LI/shading conditions [10,11]. Plant species growing in ‘marginal’ shade environments, in which transient irradiance is common, acclimate to light at the chloroplast level, whereas ‘sun’ species acclimate to irradiance changes at the leaf level and ‘shade’ species tend to lack the ability to acclimate to light at either the chloroplast or leaf level [12]. Crop plants may modulate their pigment content/ratio and enhance the light capture efficiency of photosystem II in response to LI resulting from intercropping systems [13]. Furthermore, similar to other abiotic stresses, LI can also induce the accumulation of reactive oxygen species (ROS), which then lead to plant oxidative cell damage [9]. The enhanced activities of antioxidant enzymes, such as superoxide dismutase (SOD), catalase (CAT), and peroxidase, are crucial for plant resistance to LI stress [14]. Accordingly, research into the mechanisms underlying plant LI stress tolerance has mainly focused on the growth rate, photosynthetic rate, and antioxidant system activities, with relatively few investigations on the related genes and pathways. This limits the breeding of LI-tolerant *Pyropia/Porphyra* strains.

In this study, to decipher the molecular mechanisms responsible for the acclimation of *Pyropia/Porphyra* species to LI stress, the gene expression patterns and the associated regulatory pathways induced by LI stress were analyzed in *P. haitanensis* via a combined high-throughput transcriptomic sequencing and physiological analysis. The results presented herein may be relevant for future investigations conducted to identify the genes and molecular basis of *Pyropia/Porphyra* LI tolerance, with potential implications for the breeding of new LI-tolerant strains.

## 2. Materials and Methods

### 2.1. Growth Conditions and Treatment

The experimental materials WO141-3 (LI-sensitive (LIS) strain) and 9-IV (LI-tolerant (LIT) strain) were obtained from the *Pyropia haitanensis* germplasm resource bank in Fujian province. Healthy blades (approximately 15 cm long) were selected during the prime growth stage for low-light treatments. Under normal conditions, the blades of both strains were cultured in natural seawater containing Provasoli’s enriched seawater medium at 21 °C with a 12 h light (50 μmol photons/m^2^·s)/12 h dark cycle. For the LI stress treatment, randomly selected blades were cultured in flasks (500 mL) under light (5 μmol photons/m^2^·s) for 1 and 4 days. Each treatment was completed using three biological replicates of blades cultivated in parallel in separate flasks. The culture medium was refreshed every 3 days.

### 2.2. Measurement of Pigment Contents

Blades that were 15 ± 2 cm long were selected for analysis of the contents of the following pigments: total phycobiliproteins (PBP) and the individual components phycoerythrin (PE), phycocyanin (PC), and allophycocyanin (APC) as well as chlorophyll *a* (Chl *a*). The PBP, PE, PEC, and APC levels were calculated according to a published method (Gao, 1993), which was slightly modified as described by Wang et al. (2019) [15]. The Chl *a* concentration was calculated as described by Jensen (1978) [16].

### 2.3. Determination of [Y(II)]

The effective photochemical quantum yield of photosystem II [Y(II)] of blades was measured using the Diving PAM pulse amplitude modulation apparatus (Walz, Effeltrich, Germany) [17].

### 2.4. Measurement ROS, Malondialdehyde (MDA), and Antioxidant Enzyme Activities

The ROS (H_2_O_2_) and MDA contents, as well as the CAT, SOD, and ascorbate peroxidase (APX) activities, were measured as described by Zhang et al. (2011) [18]. All experiments were completed using three biological replicates, each consisting of three technical replicates.

### 2.5. Transcriptome Analysis

Total RNA was extracted and purified using the E.Z.N.A.™ Plant RNA kit (OMEGA, Norcross, GA, USA). The purity of the extracted RNA was checked using the NanoDrop ND-1000 spectrophotometer (LabTech, Beijing, China). Only RNA samples with an A_260_/A_280_ ratio between 1.9 and 2.1 and an A_260_/A_230_ ratio greater than 2.0 were used for the subsequent analyses. Additionally, RNA integrity was assessed by 1.2% agarose gel electrophoresis. De novo transcriptome assembly and annotation (as described by Illumina) were completed by Gene Denovo Biotechnology Co., Ltd. (Guangzhou, China).

Principal component analysis (PCA) analysis is an unsupervised multi-dimensional statistical analysis method that can generally reflect the overall expression difference among samples of each group and the variation degree between samples in the group. To detect differences in gene expression patterns among samples, the “fast.prcomp” function in R (http://www.r-project.org/) was used to perform a PCA of all transcriptomic datasets of six groups (control and 1-day and 4-day LI treatments). The unigene expression levels were compared between two samples after the expression levels of all unigenes were calculated. The false discovery rate (FDR) was used to determine the threshold *p*-value for multiple tests. The following criteria were used to identify differentially expressed genes (DEGs): FDR ≤ 0.001 and a log_2_|fold-change| ≥ 1. To verify the transcription levels determined by RNA-seq, 12 unigenes were randomly selected from among the DEGs for quantitative PCR analysis, with *PhUBC* (encoding a *P. haitanensis* ubiquitin-conjugating enzyme) selected as the housekeeping gene [19]. Specific primer information is provided in Supplementary Table S1.

### 2.6. Research Route

**Figure 1:**
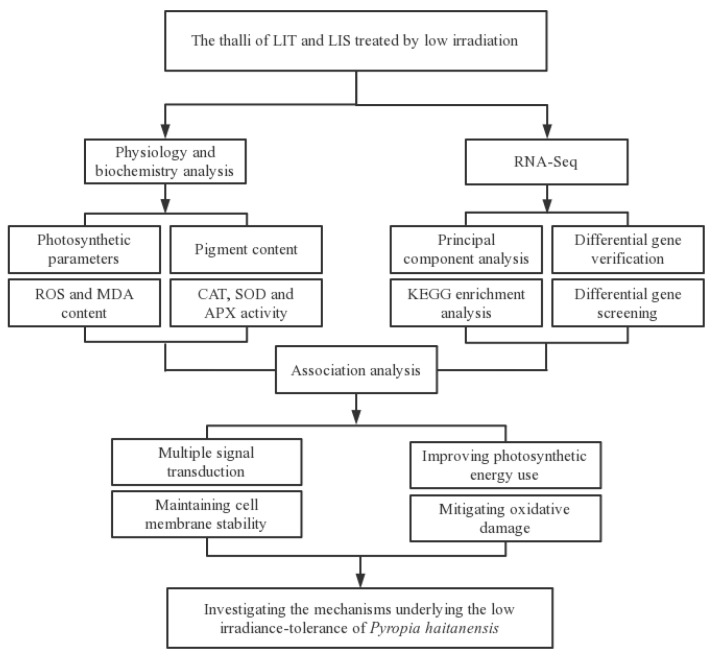
Research Route of the present study. LIT, low irradiance-tolerant strain; LIS, low irradiance-sensitive strain; ROS, Reactive oxygen species; MDA, Malondialdehyde; CAT, catalase; SOD, superoxide dismutase; APX, ascorbate peroxidase.

The following research route was formulated according to the purpose and significance of the present study (Figure 1).

### 2.7. Data Analysis

The original data obtained are first processed by Excel 2019 (16.0.10325.20082). Data were then analyzed by one-way analysis of variance (ANOVA), with a significance level of 0.05. All reported *p*-values are based on one-way ANOVA followed by least significant difference (LSD) tests unless otherwise stated.

## 3. Results

### 3.1. Changes in the Y(II) of Blades under LI Stress

**Figure 2:**
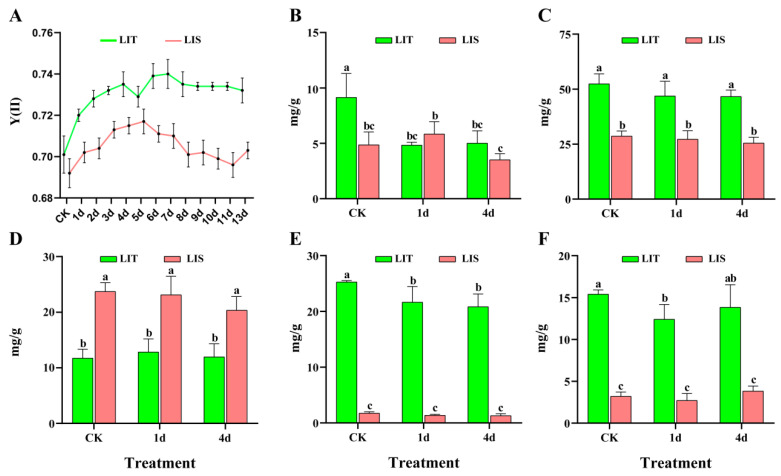
Changes in the photochemical efficiency and pigment contents in *Pyropia haitanensis* under low irradiance conditions. (**A**) photochemical efficiency of photosystem II [Y(II)]; (**B**) chlorophyll *a* (Chl *a*); (**C**) total phycobiliproteins (PBP); (**D**) phycoerythrin (PE); (**E**) phycocyanin (PC); (**F**) allophycocyanin (APC). CK, control; LIT, low irradiance-tolerant strain; LIS, low irradiance-sensitive strain. Different letters represent significant differences (*p* < 0.05).

For both strains (LIT and LIS), LI treatments significantly reduced the thalli length and weight gain (Supplementary Figure S1). The Y(II) of both strains gradually increased during the first 4 days of the LI treatment. However, Y(II) was relatively stable in the LIT strain, whereas it gradually decreased in the LIS strain after 5 days of the LI treatment. Additionally, Y(II) was significantly higher in the LIT strain than in the LIS strain in all treatment groups (Figure 2A). Accordingly, we selected days 1 and 4 as the time points for the 5 µmol photons/m^2^·s light treatment in the subsequent experiments, with normal light intensity (50 µmol photons/m^2^·s) serving as the control.

### 3.2. Changes in the Pigment Contents of Blades under LI Stress

The Chl *a* content of the LIT strain decreased significantly after 1 day of the LI treatment (*p* < 0.05) and remained relatively stable thereafter. In contrast, there was no significant change in the Chl *a* content of the LIS strain after 1 day of the LI treatment (*p* > 0.05), but there was a significant decrease after 4 days of the LI treatment (*p* < 0.05, Figure 2B). The PBP, PE, PC, and APC contents did not change significantly in the LI-treated LIS strain (*p* > 0.05, Figure 2B–E). In the LI-treated LIT strain, there were no significant changes in the PBP and PE contents (*p* > 0.05, Figure 2C,D), whereas the PC and APC contents decreased after 1 day of the LI treatment (*p* < 0.05, Figure 2E,F).

### 3.3. Antioxidant Enzyme and ROS Contents in Blades under LI Stress

**Figure 3:**
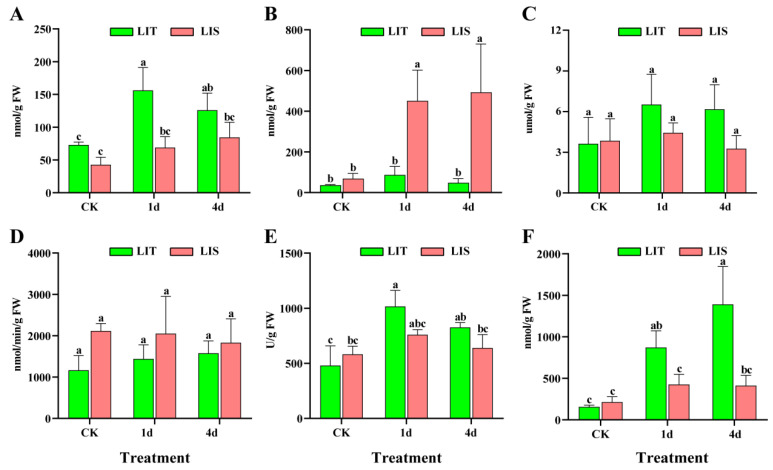
Effects of low irradiance stress on reactive oxygen species contents and antioxidant enzyme activities in *Pyropia haitanensis*. (**A**) Malondialdehyde (MDA); (**B**) superoxide anion (O_2_^−^); (**C**) hydrogen peroxide (H_2_O_2_); (**D**) ascorbate peroxidase (APX); (**E**) superoxide dismutase (SOD); (**F**) catalase (CAT). CK, control; treatment, low irradiance; LIT, low irradiance-tolerant strain; LIS, low irradiance-sensitive strain. Different letters represent significant differences (*p* < 0.05).

The MDA content of both strains increased after 1 and 4 days of the LI treatment (Figure 3A). The superoxide anion (O_2_^−^) content of the LIS strain increased significantly after 1 and 4 days of the LI treatment, but it did not change significantly in the LIT strain (*p* > 0.05, Figure 3B). The hydrogen peroxide (H_2_O_2_) and APX contents in both strains did not change significantly in response to LI stress (*p* > 0.05, Figure 3C,D). The SOD and CAT contents of the LIT strain increased significantly after 1 day of the LI treatment (*p* < 0.05), reaching levels that were higher than those of the LIS strain after 1 and 4 days of the LI treatment (*p* < 0.05, Figure 3E,F).

### 3.4. Principal Component Analysis of Transcriptome Data

**Figure 4:**
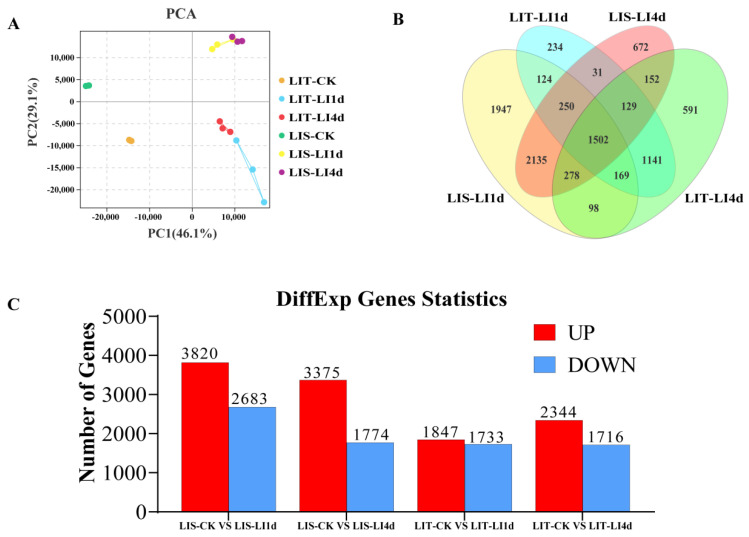
Global overview of the *Pyropia haitanensis* transcriptome under different light irradiance conditions. (**A**) Principal component analysis (PCA); (**B**) Venn diagram analysis; (**C**) number of differentially expressed genes. LIS-CK vs. LIS-LI1d, comparison between the control sample of LIS strain and the sample exposed to low irradiance for 1 day; LIS-CK vs. LIS-LI4d, comparison between the control sample of LIS strain and the sample exposed to low irradiance for 4 days; LIT-CK vs. LIT-LI1d, comparison between the control sample of LIT strain and the sample exposed to low irradiance for 1 day; LIT-CK vs. LIT-LI4d, comparison between the control sample of LIT strain and the sample exposed to low irradiance for 4 days. LIT, low irradiance-tolerant strain; LIS, low irradiance-sensitive strain. UP, up-regulated genes; DOWN, down-regulated genes.

The PCA results indicated that the three replicates of each treatment group were clustered in the same quadrant, reflecting the small variation and high repeatability of the data. However, the different treatment groups were clearly separated (Figure 4A).

### 3.5. Overall Analysis of Differentially Expressed Genes

A total of 1502 DEGs were detected in both strains after 1 and 4 days of the LI treatment on the basis of a Venn diagram analysis of all groups. There were 234 and 591 DEGs that were specific to the LIT strain after 1 and 4 days of the LI treatment, respectively, whereas there were 1947 and 672 DEGs that were specific to the LIS strain after 1 and 4 days of the LI treatment, respectively (Figure 4B).

After 1 day of the LI treatment, there were 3820 upregulated and 2683 downregulated genes in the LIS strain. In contrast, there were 1847 upregulated and 1733 downregulated genes in the LIT strain. After 4 days of the LI treatment, there were 3375 upregulated and 1774 downregulated genes in the LIS strain, which was more than the 2344 upregulated and 1716 downregulated genes in the LIT strain (Figure 4C). Additionally, the expression level trends for 10 of 12 genes were relatively consistent between the qRT-PCR and RNA-seq data, implying the RNA-seq results were reliable (Supplementary Figure S2).

### 3.6. KEGG Enrichment Analysis

**Figure 5:**
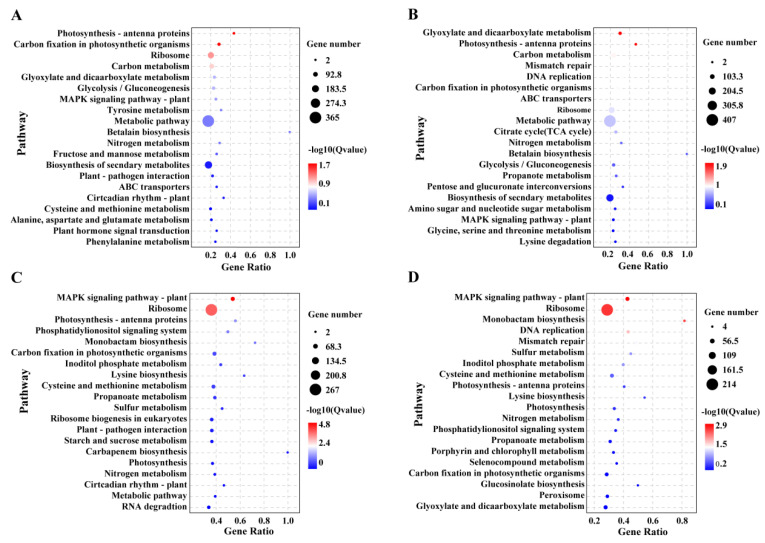
KEGG pathway enrichment analysis of the differentially expressed genes in both *Pyropia haitanensis* strains under different light irradiance stresses. (**A**,**B**) KEGG pathway enrichment analysis of the low irradiance-tolerant (LIT) strain treated with low irradiance for 1 and 4 days, respectively; (**C**,**D**) KEGG pathway enrichment analysis of the low irradiance-sensitive (LIS) strain treated with low irradiance for 1 and 4 days, respectively.

The main enriched KEGG pathways that were common to the DEGs of both strains in response to LI stress were as follows: photosynthesis, photosynthesis—antenna proteins, porphyrin and chlorophyll metabolism, phosphatidylinositol signaling system, MAPK signaling pathway—plant, and carbon fixation in photosynthetic organisms (Figure 5; Supplementary Figure S3). Additionally, the functional annotation results based on Venn diagram analysis showed that the DEGs specifically expressed in the LIT strain were mainly enriched in the metabolic pathway and TCA cycle after 4 days of LI treatment, while that in the LIS strain were mainly enriched in ribosome and protein processing in the endoplasmic reticulum (Supplementary Figure S3).

**Figure 6:**
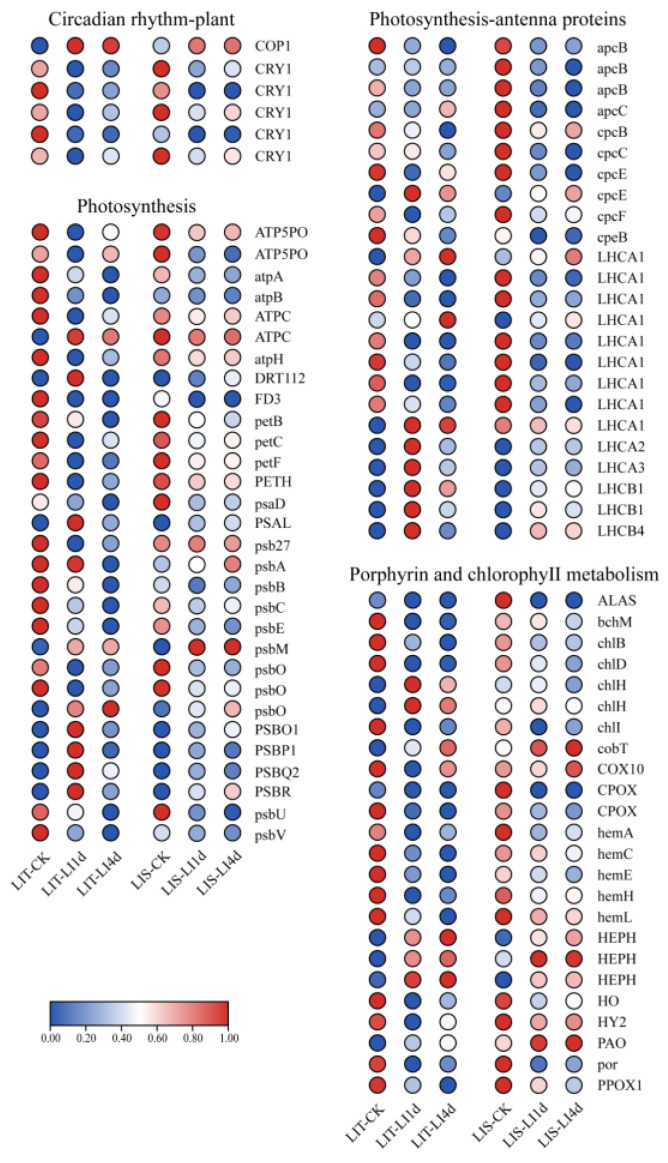
Effect of low irradiance stress on photosynthesis, photosynthesis–antenna proteins, porphyrin and chlorophyll metabolism of *Pyropia haitanensis*. LIT-CK, sample of LIT strain under normal conditions; LIT-LI1d, sample of LIT strain treated with low irradiance for 1 day; LIT-LI4d, sample of LIT strain treated with low irradiance for 4 days; LIS-CK, sample of LIS strain under normal conditions; LIS-LI1d, sample of LIS strain treated with low irradiance for 1 day; LIS-LI4d, sample of LIS strain treated with low irradiance for 4 days. LIT, low irradiance-tolerant strain; LIS, low irradiance-sensitive strain. Abbreviated gene names are listed in Supplementary Table S2.

The subsequent analysis identified one *COP1* (constitutive photomorphogenic 1) and five *CRY1* (cryptochrome 1) genes that were differentially expressed in response to the LI treatment. The *COP1* expression level was significantly upregulated only in the LIS strain under LI conditions, but the expression levels of the five *CRY1* genes were downregulated in both strains in response to the LI treatment (Figure 6). Moreover, the photosystem of the blades was also seriously affected by LI stress. In the LIT strain, the expression levels of 20 and 20 genes involved in photosynthesis were downregulated after 1 and 4 days of the LI treatment, respectively, whereas 9 and 7 photosynthesis-related genes had obviously upregulated expression levels after 1 and 4 days of the LI treatment, respectively. Among the photosynthesis-related DEGs in the LIS strain, 17 and 18 genes were obviously downregulated, and 8 and 7 genes were obviously upregulated after 1 and 4 days of the LI treatment, respectively (Figure 6). Similarly, most genes related to photosynthesis–antenna protein or porphyrin and chlorophyll metabolism had downregulated expression trends (Figure 6).

**Figure 7:**
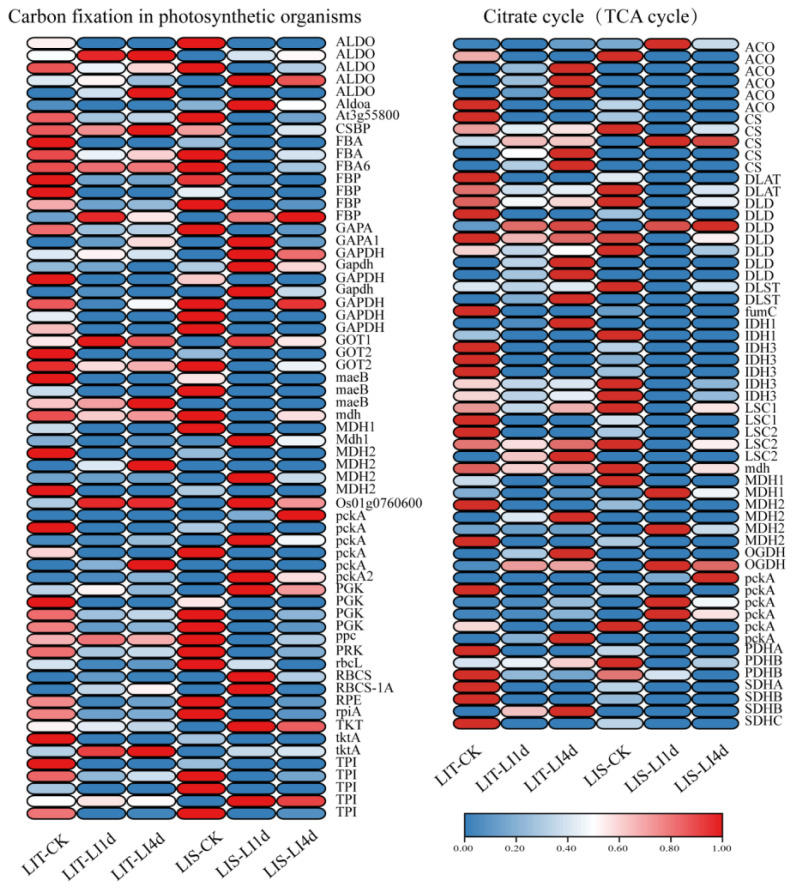
Effect of low irradiance stress on carbon fixation in photosynthetic organisms, and the citrate cycle of *Pyropia haitanensis*. LIT-CK, sample of LIT strain under normal conditions; LIT-LI1d, sample of LIT strain treated with low irradiance for 1 day; LIT-LI4d, sample of LIT strain treated with low irradiance for 4 days; LIS-CK, sample of LIS strain under normal conditions; LIS-LI1d, sample of LIS strain treated with low irradiance for 1 day; LIS-LI4d, sample of LIS strain treated with low irradiance for 4 days. LIT, low irradiance-tolerant strain; LIS, low irradiance-sensitive strain. Abbreviated gene names are listed in Supplementary Table S2.

The photosynthetic carbon fixation pathway was severely inhibited by LI stress. After 1 day of the LI treatment, 13 and 21 genes were obviously upregulated, and 36 and 35 genes were obviously downregulated in the LIT and LIS strains, respectively. After 4 days of the LI treatment, 13 and 19 genes were upregulated, and 33 and 30 genes were downregulated in the LIT and LIS strains, respectively. The expression level of the Rubisco large subunit gene (*rbcL*) decreased significantly after the LI treatment. Similarly, the genes encoding phosphoribulokinase (PRK), phosphoglycerate kinase (PGK), fructose bisphosphate aldolase (ALDO), and other key enzymes in the Calvin cycle also had downregulated expression trends following the LI treatment (Figure 7). A total of 57 DEGs were associated with the TCA cycle (Figure 7). After 1 day of the LI treatment, there were 13 upregulated genes and 33 downregulated genes in the LIT strain, whereas there were 10 upregulated genes and 34 downregulated genes in the LIS strain. After 4 days of the LI treatment, there were 20 upregulated genes and 28 downregulated genes in the LIT strain, whereas there were 10 upregulated genes and 33 downregulated genes in the LIS strain.

**Figure 8:**
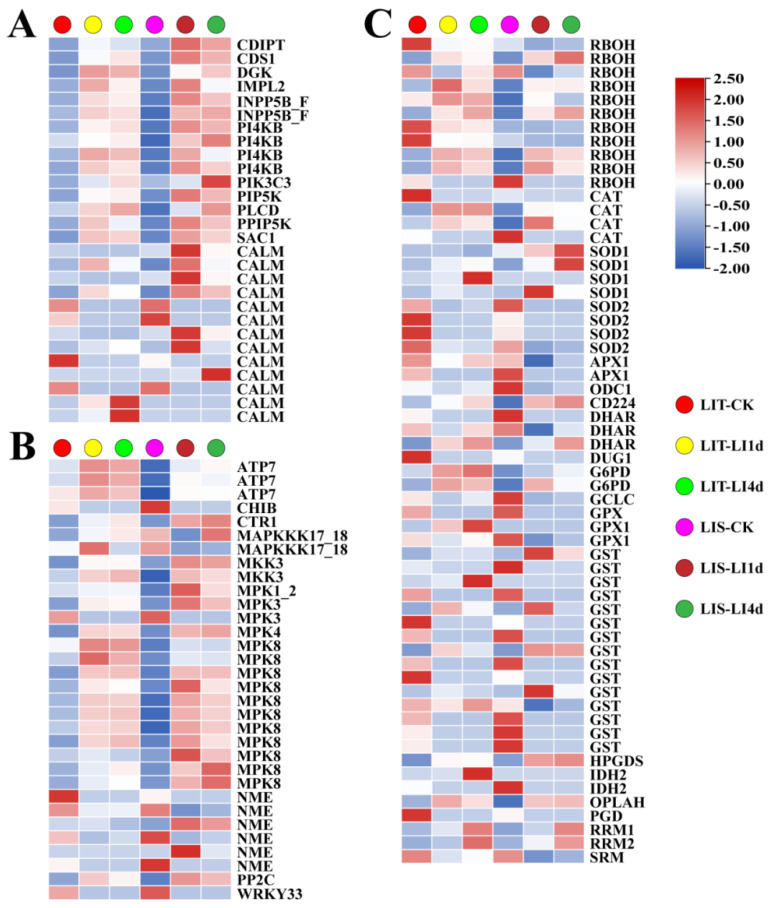
Effect of low irradiance stress on the expressions of genes related to (**A**) phosphatidylinositol signaling system, (**B**) MAPK signaling pathway–plant, and (**C**) reactive oxygen species in *Pyropia haitanensis*. LIT-CK, sample of LIT strain under normal conditions; LIT-LI1d, sample of LIT strain treated with low irradiance for 1 day; LIT-LI4d, sample of LIT strain treated with low irradiance for 4 days; LIS-CK, sample of LIS strain under normal conditions; LIS-LI1d, sample of LIS strain treated with low irradiance for 1 day; LIS-LI4d, sample of LIS strain treated with low irradiance for 4 days. LIT, low irradiance-tolerant strain; LIS, low irradiance-sensitive strain. Abbreviated gene names are listed in Supplementary Table S2.

A total of 28 DEGs related to the phosphatidylinositol (PI) signaling system were detected after LI treatment (Figure 8A), of which 20 and 20 genes were upregulated, and 7 and 7 genes were downregulated in the LIT strain after 1 and 4 days of the LI treatment, respectively. There were 20 and 21 upregulated genes and 4 and 4 downregulated genes in the LIS strain after 1 and 4 days of the LI treatment, respectively. Additionally, under LI conditions, most of the DEGs involved in the mitogen-activated protein kinase (MAPK) cascade were upregulated (Figure 8B). The major DEGs encoding MAPK kinase, including *MKK3*, *MPK8*, and *MPK4*, had upregulated expression levels following LI treatment. Among 11 respiratory burst oxidase homolog (RBOHs)-encoding genes, 6 and 6 upregulated genes and 5 and 5 downregulated genes were found in LIT strain under LI treatment for 1 and 4 days, respectively; furthermore, 6 and 5 upregulated genes and 4 and 3 downregulated genes were found in LIS strain after 1 and 4 days of LI treatment, respectively (Figure 8C). Furthermore, 8 and 8 DEGs involved in the antioxidant system (mainly encoding CAT, SOD, APX, and GST) were upregulated in the LIT strain in response to the 1-day and 4-day LI treatments, respectively. There were 9 and 10 antioxidant system-related upregulated DEGs in the LIS strain in response to 1-day and 4-day LI treatments, respectively.

## 4. Discussion

### 4.1. Effects of LI Stress on P. haitanensis Blades

Under LI conditions, photosynthetically active radiation levels may decrease, and spectral qualities may change, both of which will affect the photosynthetic activities and photomorphogenesis of plants [20]. Earlier research confirmed that COP1 accumulates in the nucleus and is active in darkness, but exposure to light induces its export from the nucleus, leading to the accumulation of transcription factors and photomorphogenesis [21,22]. The blue-light receptor CRY1 suppresses the interaction between COP1 and SUPPRESSOR OF PHYTOCHROME A and the degradation of the COP1 substrate transcription factor ELONGATED HYPOCOTYL 5 [23,24]. In the current study, the downregulated expression of *CRY1* in *P. haitanensis* decreased the repression of COP1 (Figure 6), which led to the inhibition of photomorphogenesis and decreased blade growth under LI conditions (Supplementary Figure S1). Additionally, the expression levels of most genes related to photosynthesis and energy metabolism (photosystem, photosynthesis–antenna protein, photosynthetic carbon fixation pathway, and citrate cycle) were downregulated in *P. haitanensis* blades after LI treatment. This indicates that decreases in photosynthesis and photosynthetic carbon fixation capacity ultimately lead to the insufficient synthesis of some substances. Furthermore, the effects of LI stress on *P. haitanensis* also include the excessive accumulation of ROS in cells, which leads to oxidative damage to lipids and an imbalanced cell redox state (Figure 3A). The resulting lack of energy and accumulation of ROS might be the major factors limiting *P. haitanensis* growth in response to LI stress.

### 4.2. Mechanism Underlying P. haitanensis Resistance to LI Stress

#### 4.2.1. Improved Photosynthetic Efficiency

Plants can regulate photosynthesis to adapt to LI conditions [25]. Valladares and Niinemets (2008) observed that shade-tolerant plants have higher chlorophyll contents and greater photosynthetic activities than shade-intolerant plants under LI conditions, implying the morphological features related to light perception may be optimized in shade-tolerant plants [26]. For *Pyropia/Porphyra* species, the light-harvesting pigments in the blades are phycobiliproteins (PE, PC, and APC). The PE, PC, and APC contents in the *P. haitanensis* conchocelis life cycle stage gradually increase under maturation-inducing conditions (e.g., LI as well as high temperatures and phosphorous levels). This increase helps cells maintain efficient light-harvesting activities under decreased light irradiance and duration [27]. In the present study, the PE, PC, and APC contents were higher in the *P. haitanensis* LIT strain than in the LIS strain under control and LI conditions (Figure 2). Additionally, Y(II) was significantly higher in the LIT strain than in the LIS strain at each treatment time point (Figure 2A). After 4 days of the LI treatment, Y(II) gradually decreased in the LIS strain, whereas it remained stable in the LIT strain. Accordingly, increases in light-harvesting efficiency and light-energy use may be crucial for *P. haitanensis* responses to LI stress.

#### 4.2.2. Enhanced Signal Transduction

Our previous study found that the PI signaling system is important for the perception of external stimuli and the subsequent signal transduction in *P. haitanensis* [27,28,29]. In the present study, LI treatment resulted in the upregulated expression of key genes associated with the PI signaling system in both *P. haitanensis* strains, including genes encoding PLCD, PI4KB, PIK3C3, PIP5K, and DGK (Figure 8). Phosphatidylinositol is phosphorylated by PI4Ks to produce the second messenger precursor phosphatidylinositol 4-phosphate, which is then transformed into the second messenger precursor phosphatidylinositol (4,5)-bisphosphate (PI_(4,5)_P_2_) by PIP5K; PI_(4,5)_P_2_ is converted to diacylglycerol in a reaction catalyzed by PLC, with DAG produced as a toxic lipid intermediate that is phosphorylated by DGK to produce PA (i.e., lipid second messenger) [30,31,32]. Lin et al. (2021) cloned the *P. haitanensis DGK1* gene (*PhDGK1*) and determined that its expression increases during the conchosporangia maturation stage under LI conditions and in response to short exposures to light. The expression of *PhDGK1* also increases earlier and to higher levels in the early maturing strain than in the late-maturing strain of *P. haitanensis* [27]. In addition to being a signaling lipid, PA is also an important intermediate in the lipid biosynthesis pathway because it is a precursor for the biosynthesis of the major phospholipid components in cell membranes [33]. Therefore, the results of the present study suggest that the PI signaling system is crucial for *P. haitanensis* blade responses to LI conditions.

The specific and rapid RBOH-induced ROS are critical for rapid systemic signaling activated by abiotic stresses [34,35]. H_2_O_2_ produced in response to wounding stress may function as the primary signal that activates phospholipase A2 in *P. haitanensis* blades to catalyze the degradation of membrane lipids, thereby releasing free fatty acids [36]. The Na^+^ efflux from *P. haitanensis* blades induced by hypersaline conditions is substantially inhibited by diphenyleneiodonium chloride, which is an RBOH (also known as NADPH oxidase) inhibitor [37]. Feng et al. (2022) reported that ROS produced by RBOHs could promote antioxidant enzyme activities through the ABA signaling pathway in *Pyropia yezoensis* [38]. In this study, four of eight and five of nine DEGs annotated as RBOHs had upregulated expression levels in the LIT and LIS *P. haitanensis* strains, respectively, following the LI treatment (Figure 8). The O_2_^−^ content also increased in both strains exposed to LI stress (Figure 3B). These findings imply that ROS signaling mediated by RBOHs might be critical for the induction of systemic responses that mediate *P. haitanensis* resistance to LI stress.

Recent studies confirmed that MAPK cascades are among the earliest abiotic stress-induced responses of *Pyropia/Porphyra* species [39,40]. Following the perception of extracellular stimuli, MAPK kinase kinases (MAPKKKs) activate downstream MAPK kinase (MAPKKs) via the phosphorylation of two serine/threonine residues. The activated enzymes then phosphorylate and activate MAPKs, which subsequently phosphorylate specific downstream effector proteins, including kinases and other enzymes as well as transcription factors [41,42]. Kong et al. (2021) revealed that *PyMAPK2* expression in *P. yezoensis* is rapidly induced after exposure to various abiotic stresses [43]. Our previous study also found that eight *P. haitanensis* MAPKs are phosphorylated in response to heat shock stress [44]. In the present study, MAPK cascade-related genes, 2 of 2 *MKK3* genes, 11 of 11 *MPK8* genes, and 1 of 1 *MPK4* gene, had upregulated expression levels in both *P. haitanensis* strains under LI conditions (Figure 8), suggesting that MAPK cascades are rapidly activated in *P. haitanensis* blades in response to LI stress. 

Additionally, a potential self-amplifying loop involving different signals might be important for the strong abiotic stress tolerance of *P. haitanensis*. The mechanisms underlying the feedback regulation will need to be thoroughly investigated.

#### 4.2.3. Regulation of Redox Homeostasis and Energy Supply

The excessive accumulation of ROS results in several changes, including DNA strand cleavage and oxidative stress-related lipid peroxidation [45]. In the present study, the O_2_^−^ content was higher in the LIS strain than in the LIT strain in response to the LI treatment (Figure 3B), while the activities of SOD and CAT were higher in the LIT strain than in the LIS strain. SOD and CAT are involved in maintaining ROS homeostasis in *P. haitanensis* cells [46,47,48,49]. Specifically, SOD, which contributes to the first line of cellular defense against the excessive accumulation of ROS, converts O_2_^−^ to H_2_O_2_ and oxygen [50]. In an earlier study, we used RACE technology to clone three full-length SOD-encoding genes (*PhMSD*, *PhCSD1*, and *PhCSD2*) in *P. haitanensis* while also confirming that *PhCSD1* and *PhCSD2* expression levels are highly upregulated by O_2_^−^ under desiccation conditions or at high temperatures [46]. CAT is an antioxidant enzyme that reduces H_2_O_2_, making it important for minimizing the abiotic stress-associated oxidative damage in *Pyropia/Porphyra* species [47,51]. It suggested that maintaining redox homeostasis by antioxidant enzymes is critical for *P. haitanensis* blades under LI stress, but the cultivation raft should be adjusted according to irradiation conditions to prevent oxidative damage [38].

Decreasing unnecessary energy consumption is critical for preventing cell damage in *P. haitanensis* during exposure to abiotic stress [27,52]. In the current study, the expression of 33 of 57 and 34 of 57 DEGs related to the TCA cycle was downregulated in the LIT and LIS strains, respectively, after 1 day of LI treatment (Figure 7). However, a persistent energy shortage may lead to enhanced carbohydrate metabolism and the induction of alternative pathways for the production of the energy required for key metabolic processes [53]. In accordance with this earlier finding, there were fewer upregulated TCA cycle-related genes (4) in the LIS strain than in the LIT strain (15) after 4 days of LI treatment (Figure 6), indicating that an increase in the energy supply may be another important part of the mechanism underlying the LI stress tolerance of the LIT strain. The increased pigment contents in the LIT strain can increase the photosynthetic efficiency of the blades, thereby increasing more energy supply than the LIS strain. 

## 5. Conclusions

The main detrimental consequences of exposure to LI conditions during *P. haitanensis* cultivation includes insufficient light energy intake, insufficient energy supply, and the accumulation of ROS, which will adversely affect blade growth. In order to resist LI stress, potential self-amplifying signal transduction pathways might activate downstream systemic responses, ultimately leading to increased photosynthetic efficiency and antioxidant enzyme activities as well as LI stress resistance. The present result is useful for elucidating the mechanisms enabling seaweed to cope with LI stress and provides new ideas for breeding new varieties.

## Data Availability

Read data are available from the Sequence Read Archive (SRA), accessible through NCBI BioProject ID PRJNA826655.

## Supplementary Materials

The following supporting information can be downloaded at: https://www.mdpi.com/article/10.3390/life13020481/s1, Figure S1: The growth characteristics of thalli of different *P. haitanensis* strains under low irradiance (LI) conditions. A–D represent the length changes, the growth rate of length, the weight changes, and the increase rate of weight of the thalli, respectively; LIT, LI-tolerance strain; LIS, LI-sensitive strain; CK, control. Different letters represent significant differences among the thalli of same strain under LI conditions (*p* < 0.05); Figure S2: validation of RNA sequencing data on 12 selected genes under low irradiance 5 μmol/(m^2^·s) treatment; Figure S3: KEGG pathway enrichment analysis of the different expressed genes that co-expressed and specifically expressed in two *P. haitanensis* strains under different light irradiance stresses. A and B, KEGG pathway enrichment analysis of specifically expressed genes in the low irradiance-tolerant (LIT) strain treated with low irradiance for 1 and 4 days, respectively; C and D, KEGG pathway enrichment analysis of specifically expressed genes in the low irradiance-sensitive (LIS) strain treated with low irradiance for 1 and 4 days, respectively. E, KEGG pathway enrichment analysis of co-expressed genes in both strains under different light irradiance stresses. Table S1: The names and sequences of primers used in the RT-PCR experiment; Table S2: Abbreviated gene names of differentially expressed genes.
