# Supplementary material for: Investigating the Mechanisms Underlying the Low Irradiance-Tolerance of the Economically Important Seaweed Species Pyropia haitanensis
Source: Life (Basel). 2023 Feb 9;13(2):481. doi: 10.3390/life13020481 (PMC9965670; doi:10.3390/life13020481)
Supplement: Supplementary file 1 [file life-13-00481-s001.zip › Supplementary Figures.pdf]

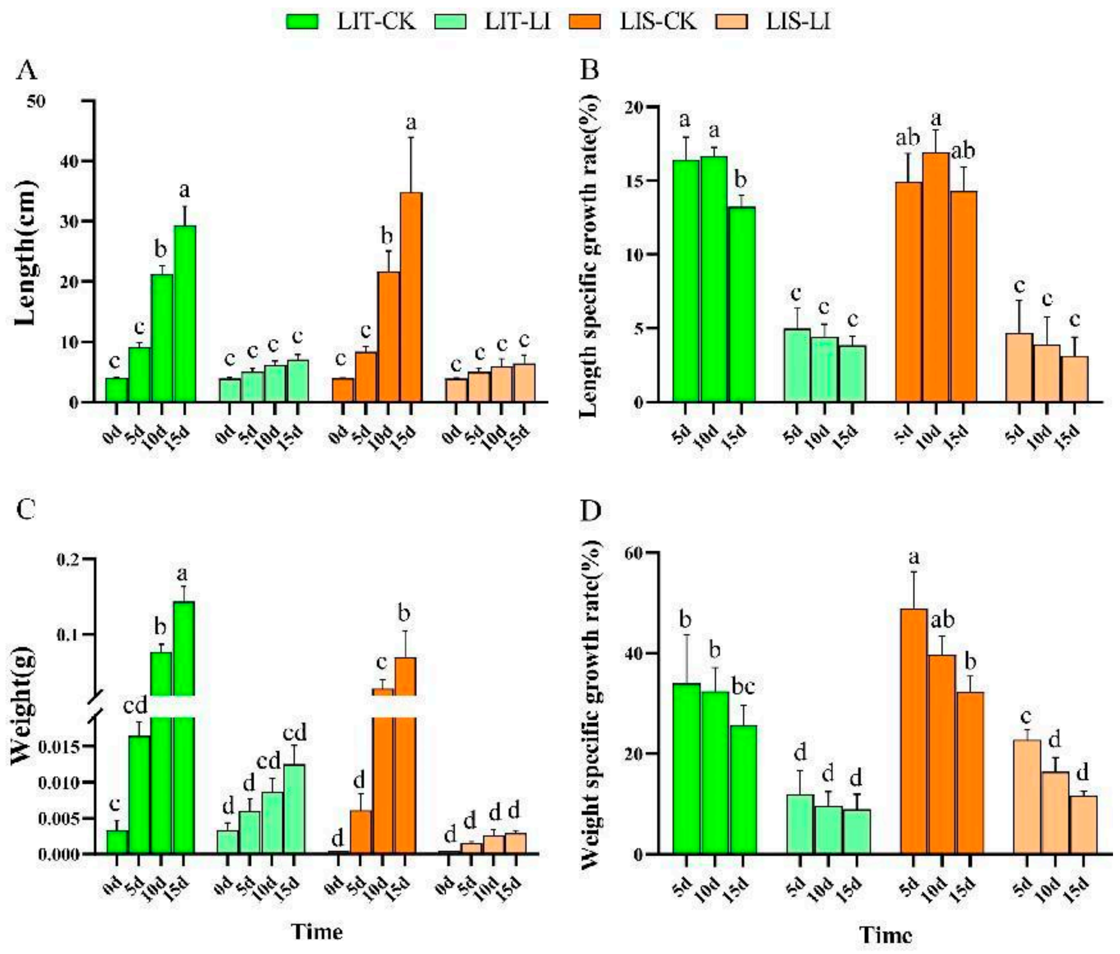

Figure S1 The growth characteristics of thalli of different *Pyropia haitanensis* strains under low irradiance (LI) conditions. A - D represent the length changes, the growth rate of length, the weight changes, and the increase rate of weight of the thalli, respectively; LIT, LI-tolerance strain; LIS, LI-sensitive strain; CK, control. Different letters represent significant differences among the thalli of same strain under LI conditions ($P < 0.05$)

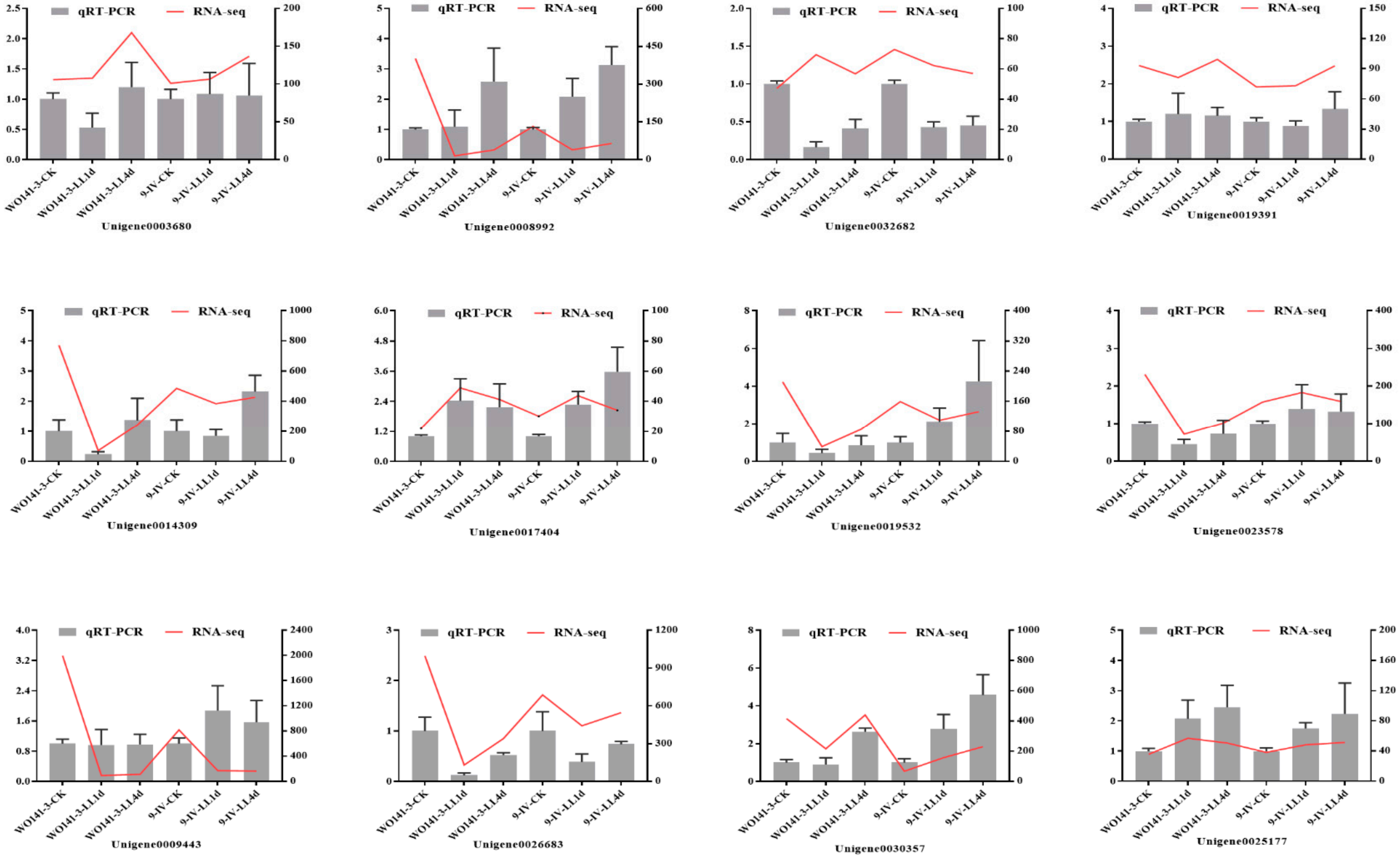

Supplementary Figure S2 qPCR validation of RNA sequencing data on 12 selected genes under low irradiance 5 μmol/(m^2·s) treatment

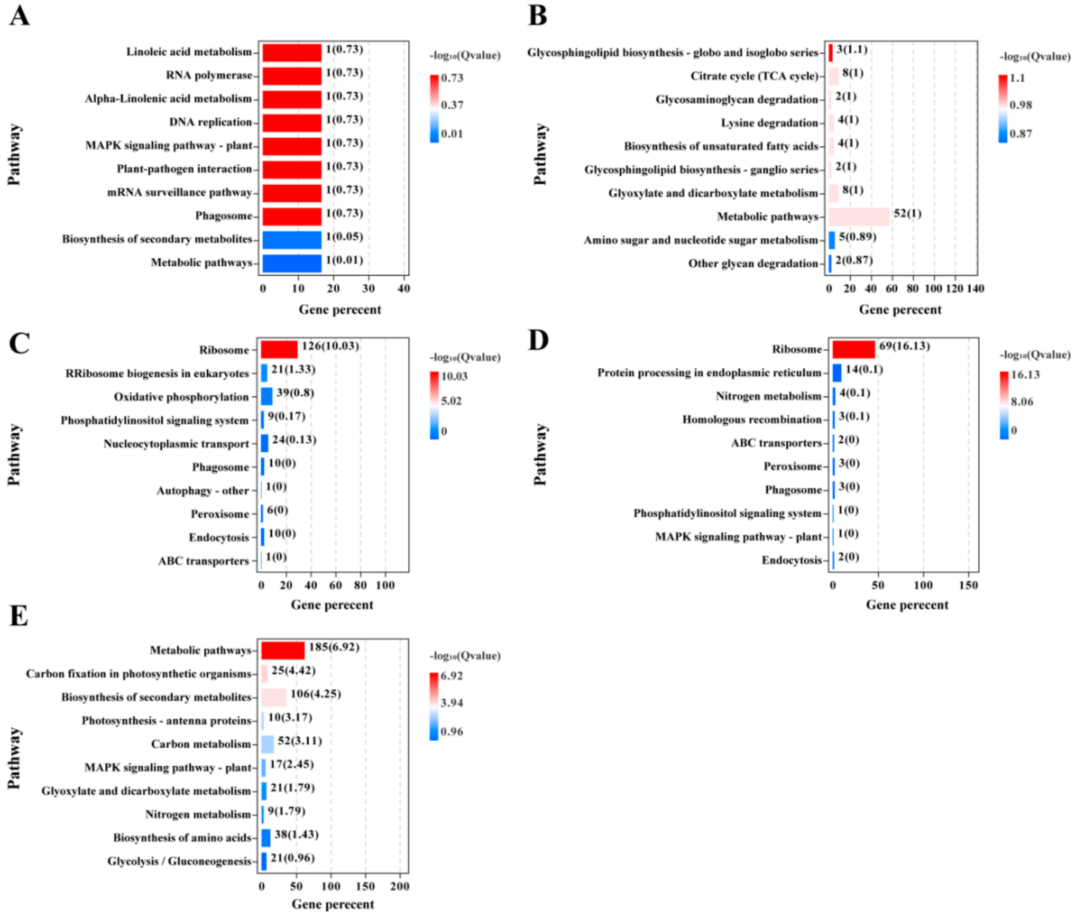

Figure S3 KEGG pathway enrichment analysis of the different expressed genes that co-expressed and specifically expressed in two *Pyropia haitanensis* strains under different light irradiance stresses. A and B, KEGG pathway enrichment analysis of specifically expressed genes in the low irradiance-tolerant (LIT) strain treated with low irradiance for 1 and 4 days, respectively; C and D, KEGG pathway enrichment analysis of specifically expressed genes in the low irradiance-sensitive (LIS) strain treated with low irradiance for 1 and 4 days, respectively. E, KEGG pathway enrichment analysis of co-expressed genes in both strains under different light irradiance stresses.
