# Supplementary material for: Investigating the Mechanisms Underlying the Low Irradiance-Tolerance of the Economically Important Seaweed Species Pyropia haitanensis
Source: Life (Basel). 2023 Feb 9;13(2):481. doi: 10.3390/life13020481 (PMC9965670; doi:10.3390/life13020481)
Supplement: Supplementary file 1 [file life-13-00481-s001.zip › Supplementary Table S1.pdf]

Table S1 The names and sequences of primers used in the RT-PCR experiment

Gene ID	Primer sequences
Unigene0003680	F: GGTGGCGATGGGTGACAACG<br>R: TCGGATCCTTGAGGCCGAGT
Unigene0008992	F: GCGTCATGACTCGGCGTACT<br>R: GGCGAGAAGTCGTCGATCCG
Unigene0009443	F: TCGGAGGAAGCCAAGGTCCA<br>R: CGCCATGGGCACGTTGTACT
Unigene0032682	F: CTCTCTTCCTCCCAGCCAAC<br>R: CGTACGTGAGCATCTTGACC
Unigene0019391	F: GTACGGCATTCCAGTGTCGG<br>R: CTGATGCGGTCTGCTCCTTG
Unigene0014309	F: GCAGCCGATCATGGAGTCGT<br>R: CACCGAGTGCTTGGCCTTGT
Unigene0017404	F: GCCGGTGTACGTGCTCATCA<br>R: AGCGAGCACGTGTTGACGAT
Unigene0019532	F: GGCCGTTTGGGACCGAGAG<br>R: AAGGCAAACACCTGGCCACA
Unigene0023587	F: GCAGGGACCATTGGCAACCT

	R: CCACCGACGTATGGGTGAGC
Unigene0025177	F: GGCAGAACAAGACGGGTGAG
	R: GGAAGAGCGACATGACCGAC
Unigene0026683	F: GACGATTGCGTCGGACCTGT
	R: CCGTATCGTACGCCGTCTCG
Unigene0030357	F: CCAAGCCGCTGGTAGAGTTC
	R: TAGTTGACGGCCAGAACCAC
UBC	F: TCACAACGAGGATTTACCACC
	R: GAGGAGCACCTTGGAAAGG
